# Dataset of global extreme climatic indices due to an acceleration of ice sheet melting during the 21st century

**DOI:** 10.1016/j.dib.2019.104585

**Published:** 2019-09-28

**Authors:** Dimitri Defrance

**Affiliations:** SYSTEM, Univ Montpellier, INRA, Montpellier SupAgro, CIRAD, CIHEAM, Montpellier, France

**Keywords:** Ice sheet contribution, Climate change, Extreme events, Temperature, Precipitation, Global climate model

## Abstract

This article describes extreme indices maps (Data Cube, raster X Time) for different scenarios with a more important contribution to the sea level rise from Greenland and/or Antarctica during the 21st century under the Representative Concentration Pathway (RCP) 8.5 emission scenario. The indices are produced annually and globally with a resolution of 0.5° × 0.5° from 1951 to 2099. The data were generated by simulating daily maximum and minimum temperature and precipitation from the IPSL-CM5A-LR model from Coupled Model Intercomparison Project Phase 5 (CMIP5). These climatic data are unbiased and downscaled to the 0.5°x0.5 scale with the Cumulative Distribution Function transform (CDFt) and EWEMBI dataset compiled to support the bias correction of climate input data for ISIMIP. Finally, each extreme indice is computed on the unbiased data on each grid cell on all continents.

**Table:** Specifications Table

Subject	Climate change and extreme events
Specific subject area	Evolution of extreme events due to a high contribution of freshwater from ice sheets.
Type of data	Data Cube (Raster X Time) in NetCDF
How data were acquired	Climatic simulation with Global Circulation Model using current and projected gasses emissions
Data format	Raw
Parameters for data collection	Geographic Coordinate System WGS1984 with 0.5 × 0.5 spatial resolution
Description of data collection	Unbiased Temperature and Precipitation data from IPSL-CM5A-LR climate model to compute 16 extreme climatic indices
Data source location	Global scale (Entire World)
Data accessibility	Repository name: Mendeley Data identification number: 10.17632/fbsdj87gjg.1 Direct URL to data: https://doi.org/10.17632/fbsdj87gjg.1

**Table:** 

**Value of the Data** • Data consists of 16 climatic indices computed on numerical simulation of future climate, taking into account the acceleration of the ice sheets melting during the 21st century. • The novelty of this data is the fact that this acceleration is usually not taken into account in climate projections (e.g. in IPCC reports) and that it elaborates upon raw climate data by computing indices useful for impact studies. • These indices, describe the frequency and intensity of extreme events (heat or cold waves, dry or wet spells) at the annual scale and with a spatial resolution of 0.5° × 0.5° globally • Data are useful for non-climate change experts, e.g. insurances companies, policy makers, or researchers who wish to integrate this type of data into multidisciplinary studies such as disaster risk management, food security. • This dataset was obtained from only one General Circulation Model (IPSL-CM5A-LR), but it explores different scenarios of ice sheet melting (Greenland, Antarctica or both, with different levels).

## 1 Data

The data consist of 16 annual extreme indices on the entire world and given in a geo-tiff raster datasets. These indices depend on daily precipitation or daily temperature simulated by the General Circulation Model (GCM) IPSL-CM5A-LR used in the Coupled Model Intercomparison Project Phase 5 (CMIP5). A historical run and seven different climatic scenarios are simulated between 1951 and 2006 (historical) and between 2006 and 2099 (climatic scenarios). The first climatic scenario is the RCP8.5 baseline scenario from the Intergovernmental Panel on Climate Change (IPCC) corresponds to a global temperature increase of ∼5 °C with respect to the pre-industrial level. The six other scenarios are superimposed on the RCP8.5 scenarios with an input of freshwater from Greenland and/or West-Antarctica added in the ocean. The amount of added freshwater corresponds to 1, 1.5 or 3 m of sea level rise.

**Fig. 1:**
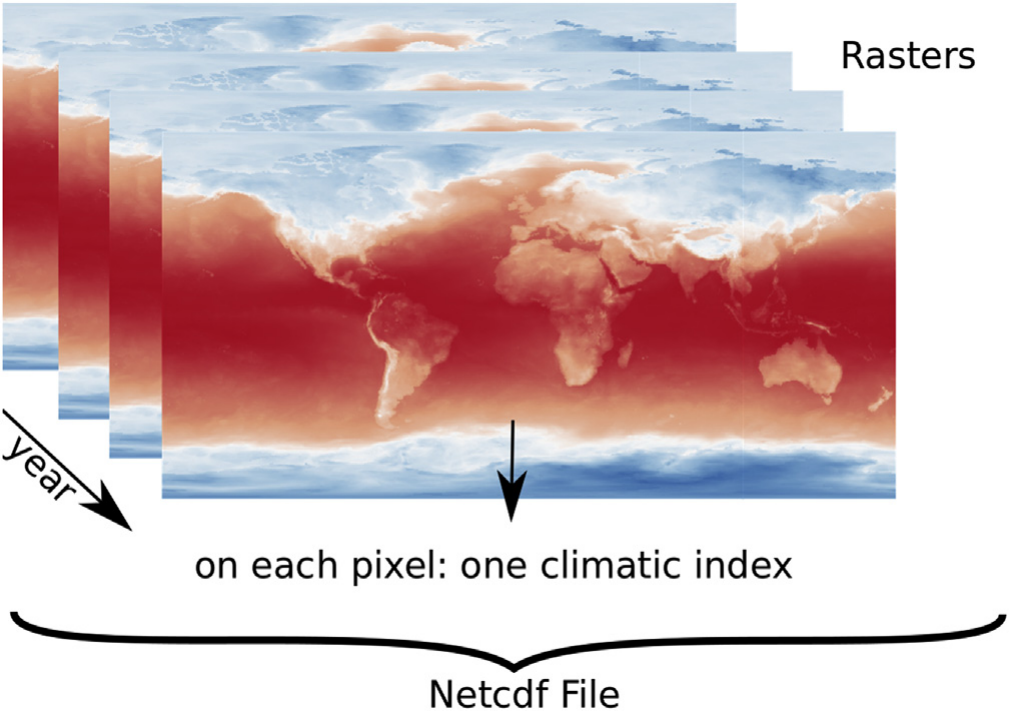
Structure of climatic index (raster map X year).

The results are seven annual time series of the 16 indices across the world with a 0.5° × 0.5° resolution (∼50 × 50 km at the equator) and an historical run from 1951 to 2005. The different climate indices characterize the extreme events related to temperature and precipitation and have been determined by Expert Team on Climate Change Detection and Indices (ETCCDI). For each grid cell, we have seven different annual evolutions of each index by the end of 21st century (Fig. 1.). It can be accessed via https://doi.org/10.17632/fbsdj87gjg.1.

## 2 Experimental design, materials, and methods

To calculate the different extreme climatic indices under an acceleration of ice-sheet melting, three steps are necessary:

1) Climatic simulation (precipitation and temperature) obtained by a GCM,
2) Unbiased temperature and precipitation time series,
3) Calculation of 16 climatic indices.

### 2.1 Climatic simulation

All the experiments are carried out with the coupled atmosphere-ocean model IPSL-CM5A-LR [1] used in international intercomparison projects of climate models (CMIP5) whose results are reported by the IPCC. The atmospheric component (LMDZ) has a spatial resolution of 3.75° over 1.875° in longitude and latitude respectively with 39 vertical levels; the oceanic component (NEMO) uses an irregular grid with a nominal resolution of 2° and a finer resolution in the latitude of 0.5° in the equatorial ocean and on 31 vertical levels (For details see Ref. [1]).

The climate scenario that is considered in this dataset and which serves as a baseline scenario is the RCP8.5 for the period 2006 to 2100. It corresponds to the most pessimistic evolution of the climate by the IPCC and leading to an average increase of temperature of about 5 °C compared to that of the pre-industrial era. In this scenario, a flow of freshwater corresponding to a sea level rise of 1 m, 1.5 m and 3 m (0.11 sverdrups (Sv), 0.22 Sv, 0.34 Sv and 0.68 Sv, 1Sv = 10^6^ m^3^/s) is added from 2020 to 2070. Three freshwater flow scenarios are considered:

• the freshwater comes from Greenland (GrIS scenario) and is therefore introduced into the Atlantic Ocean [45°N-65°N, 45°W-5°E];
• the freshwater comes from West Antarctic (WAIS scenario) and is therefore introduced around West Antarctica up to 60°S;
• the freshwater comes from both ice sheets (GrWAIS scenario) and is therefore introduced simultaneously into the Atlantic Ocean [45°N-65°N, 45°W-5°E] and around West Antarctica up to 60°S.

Locations of freshwater addition are chosen to produce a rapid model response. Freshwater discharges are therefore placed in deep-water formations in the North Atlantic for the GrIS and the GrWAIS scenarios and in the western part of the Southern Ocean for the WAIS and GrWAIS scenarios. For the GrWAIS scenario the contribution of each ice sheet is identical. The choice of high volumes of fresh water (0.11 Sv–0.68 Sv) is justified by the fact that current climate models do not seem sufficiently sensitive to freshwater inputs [2]. These high values make it possible to better evaluate the potential impacts on the thermohaline circulation in the Atlantic.

### 2.2 Unbiased temperature and precipitation time series

Several studies have shown that climate models, despite improvements over the years, are subject to significant biases for precipitation and current temperature. The study by Ref. [1] shows that the IPSL-CM5A-LR has for example a cold bias in West Africa and a warm bias on the Gulf of Guinea which prevents a good estimate of monsoon rains. All of the global temperature and precipitation biases are described in this article for more detail.

To circumvent this disadvantage, we applied a statistical method to improve the precipitation and temperatures simulated by the IPSL-CM5A-LR in the world. This method, called “Cumulative Distribution Function transform” (CDF-t) was originally developed by Ref. [3] and has been successfully applied in many climate studies (e.g. Ref. [4]). This is a variant of the “Quantile Mapping Approach” (e.g. Ref. [5]), which is detailed in the article by Ref. [6]. The Quantile Mapping Approach technique involves associating with a modeled precipitation value (or other climatic variables) a precipitation value in the control distribution (i.e. the observations or the reanalysis dataset), so that cumulative distributions (modeled and reference values) are equivalent [7].

The CDF-t method goes even further and takes into account the climate change signal in the corrected dataset. This is made possible by the assumption that there is a mathematical transformation to translate the cumulative distribution function of the modeled variables to be corrected into the cumulative distribution function representing climate variables from the reference dataset (for the period history or calibration) or corrected values (for the future).

The climate simulations have been corrected with respect to the interpolated EWEMBI climate reanalysis at a spatial resolution of 0.5° × 0.5° [8], used as a reference. Here, the so-called “calibration” period covers the 34-year period from 1979 to 2013, while the “projection” period covers the 94-year period from 2006 to 2099. The raw data (temperature and precipitation), from the GCM IPSL-CM5A-LR, have a resolution of 3.75° × 1.875° and are interpolated on the 0.5° × 0.5° resolution (as EWEMBI) using a bilinear approach for temperatures and using a “nearest neighbour” approach for precipitation. Secondly, the CDF-t approach is applied on the daily time series for the different climate variable, grid point by grid point. Finally, bias corrected daily rasters are obtained for precipitation and for minimum or maximum temperature.

### 2.3 Calculation of 16 climatic indices

In order to better characterize global changes related to the melting of Greenland and Antarctica, climate indices other than precipitation totals and mean annual temperatures are computed. On Table 1, Table 2, these indicators are based on daily surface data: minimum temperature (TN), maximum temperature (TX) (Table 1) and precipitation (PR) (Table 2). The 16 climatic indices come from a set of indicators defined by the ETCCDI supported by the CLIVAR project (http://www.clivar.org/) and are well described in Refs. [9,10] and on the ETCCDI website (http://etccdi.pacificclimate.org/list_27_indices.shtml). The different indices are calculated year by year on each grid cell (0.5° × 0.5°) around the world by Climate Data Operator (CDO) and Climate indices of daily temperature and precipitation extremes library.

**Table 1:** 8 Climatic indices based on the maximum (TX) and minimum (TN) daily temperature.

Label	Index name	Index definition	Units
TNmin	Annual minimum temperature	Let TNmin be the daily minimum temperature in the year j. The minimum daily minimum temperature is then: TNminj = min(TNj)	°C
TXmax	Annual maximum temperature	Let TXmax be the daily maximum temperature in the year j. The maximum daily maximum temperature is then: TXmaxj = max(TXj)	°C
FD	Number of frost days	Let TN be the daily minimum temperature on day i in year j. Count the number of days where TNij <0 °C	days
ID	Number of icing days	Let TX be the daily maximum temperature on day i in year j. Count the number of days where TXij <0 °C	days
TR	Number of tropical nights	Let TN be the daily minimum temperature on day i in year j. Count the number of days where TNij >20 °C	days
SU	Number of summer days	Let TX be the daily maximum temperature on day i in year j. Count the number of days where TXij >25 °C	days
WSDI	Warm speel duration index	Let TXij be the daily maximum temperature on day i in year j and let TXin90 be the very hot threshold corresponding to 90th percentile centered on a 5 day window for the historical period 1976–2005. Then each year, the number of warm period with at least 6 consecutive days: TXij > TXin90 is summed.	days
CWDI	Cold wave duration index	Let TNij be the daily minimum temperature on day i in year j and let TNin10 be the very cold threshold corresponding to 10th percentile centered on a 5 day window for the historical period 1976–2005. Then each year, the number of cold period with at least 6 consecutive days: TNij < TNin10 is summed.	days

**Table 2:** 8 Climatic indices based on the daily precipitation.

Label	Index name	Index definition	Units
PRCPTOT	Total wet-day precipitation	Let PRij be the daily precipitation amount on day i in year j. If I represents the number of days in j, then: PRCPTOTj = $\sum_{n=1}^{I}$ PRij	mm
R1mm	Annual count of wet days	Let PRij be the daily precipitation amount on day i in year j. Count the number of days where PRij >1 mm	days
R10mm	Annual count of days when PRCP≥ 10mm	Let PRij be the daily precipitation amount on day i in year j. Count the number of days where PRij >10 mm	days
R20mm	Annual count of days when PRCP≥ 20mm	Let PRij be the daily precipitation amount on day i in year j. Count the number of days where PRij >20 mm	days
SDII	Simple precipitation intensity index	Let PRwj be the daily precipitation amount on wet days, PR > = 1 mm in year j. If W represents number of wet days in j, then: SDIIj = ($\sum_{w=1}^{w}$PRwj)/W	mm
RX5day	maximum consecutive 5-day precipitation	Let PRkj be the precipitation amount for the 5 day interval ending k, in year j. Then maximum 5 day values for period j are: RX5dayj = max (PRkj)	mm
CDD	Maximum length of dry spell, maximum number of consecutive days with RR < 1mm	Let PRij be the daily precipitation amount on day i in year j. Count the largest number of consecutive days where PRij <1 mm	days
CWD	Maximum length of wet spell, maximum number of consecutive days with RR ≥ 1mm	Let PRij be the daily precipitation amount on day i in year j. Count the largest number of consecutive days where PRij >1 mm	days
